# Exploring the Connection between the Occurrence and Intensity of “Grubby” Defect and Volatile Composition of Olive Oil

**DOI:** 10.3390/foods12244473

**Published:** 2023-12-14

**Authors:** Karolina Brkić Bubola, Igor Lukić, Marin Krapac, Olivera Koprivnjak

**Affiliations:** 1 Institute of Agriculture and Tourism, Karla Huguesa 8, 52440 Poreč, Croatia; igor@iptpo.hr (I.L.); marin@iptpo.hr (M.K.); 2 Faculty of Medicine, University of Rijeka, Braće Branchetta 20, 51000 Rijeka, Croatia; olivera.koprivnjak@uniri.hr

**Keywords:** olive fruit fly, olive oil, volatile compounds, sensory characteristics, “grubby” defect

## Abstract

In order to investigate the relationship between the occurrence of the “grubby” sensory defect caused by olive fruit fly (*Bactrocera oleae* (Rossi)) infestation and the resulting volatile composition, virgin olive oils were extracted from olives of the Leccino cultivar with 0%, 50%, and 100% olive fly infestations and subjected to analysis of the basic chemical quality parameters, fatty acids and volatiles, and sensory analysis by the Panel test. A 100% olive fly infestation reduced the basic chemical quality of the oil, while the fatty acid composition was not affected in any case. The overall sensory quality score and intensity of the positive sensory attributes decreased, while the intensity of the “grubby” defect increased proportionally to the degree of infestation. The occurrence and intensity of this defect were clearly causally related to the concentrations of 3-methylbutanal, 2-methylbutanal, β-ocimene, ethyl 2-methylbutyrate, dimethyl sulfoxide, 4-methyl-5H-furan-2-one, α-farnesene, 6-methyl-5-hepten-2-one, 1-octanol, *E*-2-nonen-1-ol, benzeneacetaldehyde, heptanal, and octanal, implying that the perception of “grubby“ comes from their joint contribution to the overall olive oil flavour. In addition to contributing to the understanding of the chemical origin of “grubby”, the results obtained could potentially be used to develop strategies to support sensory analysis in the classification of olive oil quality and the confirmation of the presence of this sensory defect in oil samples.

## 1. Introduction

Virgin olive oil (VOO) is still the only example of a food whose quality assessment includes an official method of sensory analysis for the purpose of commercial classification at the international level [1]. The sensory analysis method (Panel test) is based on the identification and intensity determination of the fruitiness as well as of the dominant pre-descripted sensory defect [2]. In recent years, there has been a great interest in finding and elucidating the relationship between the sensory characteristics of virgin olive oil and the volatiles that determine them, which would facilitate and improve the performance of the Panel test. The current focus is on the selection of relevant volatile markers that are useful to: (i) identify or confirm the presence of the most common sensory defects in virgin olive oils [3,4,5], (ii) be included in formulated reference materials for sensory assessor training and panel reliability verification [6], and (iii) classify extra virgin olive oils into green or ripe fruity types [7]. Regarding off-flavours, attention is mainly focused on those included in the main list of negative sensory attributes (defects) of the official method, i.e., “fusty/muddy sediment”, “musty/humid”, “winey-vinegary/acid-sour” [4,5,6,8,9,10], “rancid” [3,6,11], and “frostbitten olives” [12,13]. 

Similar studies on negative sensory attributes included in the secondary list of the official method, such as “heated/burnt”, “hay/wood”, “vegetable water”, or “grubby”, are much less represented. The defect “grubby” is described as a “flavour of oil obtained from olives which have been heavily attacked by the grubs of the olive fly (*Bactrocera oleae*)” [2]. This olive fruit pest is considered the most damaging in the Mediterranean region. The larvae, which feed on the tissue inside the drupe, have negative effects on various aspects of oil quality, the intensity of which is related to the extent of tissue damage, the percentage of infested fruits, the ripeness level of the fruits, the time and conditions of fruit storage, and the intrinsic characteristics of a particular olive cultivar [14,15]. Angerosa et al. [16] were among the first to investigate the relationship between olive fly infestation intensity and the presence of specific volatile compounds in virgin olive oil. After that, there were few studies on this topic [17,18,19]. As for the influence of olive fly infestation on the enzymatic synthesis of volatile compounds from the lipoxygenase (LOX) pathway, one observation common to most of the studies mentioned was associated with an increase in C6 alcohol levels. Among C6 aldehydes, an increase in hexanal [16,18] and a decrease in *Z*-3-hexenal level [19] were highlighted, while there was no agreement on the effects on *E*-2-hexenal, which is the predominant volatile product of the LOX pathway. The activity of enzymes involved in the formation of these substances is triggered by the destruction of cell structures. However, Alagna et al. [20] suggested that mechanical tissue damage by larval feeding is not the only cause of changes in the volatile profile of olive fruit and supposed that there are additional mechanisms of interaction between plants and insects. In addition, it is known that a greater exposure of internal tissues to microbial and oxygen activity due to damage to the exocarp can lead to various fermentative processes, accompanied by an increase in the levels of ethanol and branched-chain alcohols from amino acid metabolism [16,17], as well as ethyl acetate [16,19] in the volatile fraction of the oil. This is consistent with the fact that the most frequently found defects in oils from olive fly-infested olives are “fusty” [16,17,21], “musty” [17,21,22], and “rancid” [17,21,23], while “grubby” was only occasionally noted [17,23] or not reported. The sensory perception of the defect “grubby” is not yet clearly defined, as it refers to a combination of taste and odour characteristics that can rarely be independent of oxidation- and fermentation-related changes and associated volatile products. Moreover, it has been previously shown that sensory defects can sometimes be absent even in the case of 100% olive fly infestation, which translates only into a decrease in the intensity of positive sensory characteristics of olive oil [18].

The present study aimed primarily to investigate the relationship between the intensity of the defect “grubby” and the corresponding composition of volatile compounds of virgin olive oil as a result of varying degrees of olive fruit fly infestation. The intention was to extract the most potent volatile markers capable of distinguishing defective olive oils produced from infested fruits from those made from healthy ones and, in this way, to gain a deeper understanding of the causes of this common defect. Clearer detection and identification, as well as better characterization of the defect “grubby”, were achieved by processing the olives under controlled conditions without prior fruit storage in order to minimize interferences of oxidative and fermentative origin related to fruit storage. An additional objective of this study was to evaluate the effects of olive fly infestation on the fatty acid composition of virgin olive oil and on the basic properties of the corresponding olive pastes.

## 2. Materials and Methods

### 2.1. Preparation of the Samples of Virgin Olive Oil

Olive fruits of the Leccino cultivar, grown in the Istria region of Croatia, were harvested by hand at a ripening index (RI) of 3.0 (purple epidermis) from the same tree [24]. Fruits were divided into two groups: group L-100% represents fruits with at least one olive fly puncture or exit hole, while group L-0% represents healthy fruits. The approximate ratio between fruits with exit holes and those with punctures was 80% and 20% of the total number of infested fruits, respectively. A third group (L-50%) was prepared by mixing the fruits from the L-100% and L-0% groups in equal proportions to simulate an intermediate level of fruit infestation. Each group of olive fruits was processed in triplicate within 24 h after harvesting using an Abencor system (MC2 Ingenieria y Sistemas, Seville, Spain). Olive fruits (2 kg) were crushed with a hammer crusher and olive pastes were obtained by malaxation at 24 ± 1 °C for 45 min followed by centrifugation at 3500 rpm for 60 sec to extract the virgin olive oils. The VOOs obtained were decanted and stored in dark bottles at room temperature until analysis.

### 2.2. Determination of the Water and Oil Content 

To determine the water content gravimetrically, olive paste samples (50 g) were collected and dried at 80 °C to a constant weight. The dried olive pastes were then stored at –18 °C until analysis. Frozen dry olive pastes were carefully ground with a mortar and pestle and dried again before extraction. The theoretical oil content was determined using the Soxtec Avanti 2055 instrument (Foss Tecator, Höganäs, Sweden) according to the method described by Brkić et al. [25].

### 2.3. Determination of the Quality Parameters 

The quality parameters, free fatty acids (FFA) [26], peroxide value (PV) [27], and spectrophotometric indices (K_232_, K_268_, and ΔK) [28] in the oils were determined according to the International Olive Council (IOC) analytical methods recommended by the European Commission [29]. 

### 2.4. Determination of the Fatty Acid Methyl Esters (FAME)

FAMEs were determined according to the IOC method [30] recommended by [29] using a Varian 3350 gas chromatograph (Varian Inc., Harbour City, CA, USA) equipped with an Rtx-2330 capillary column (Restek, Bellefonte, PA, USA) and a flame-ionization detector (FID). Identification was based on retention times with respect to the standard FAME mixture (Sigma, Roedermark, Germany) and according to the reference method. The relative amount of each fatty acid was expressed as a proportion (%) of the total peak area of fatty acids.

### 2.5. Sensory Analysis

The sensory analysis of VOO was carried out according to the IOC method [2] by the IOC-approved panel for sensory assessment of VOO. The panel consisted of eight assessors (five women and three men) trained in the sensory analysis of VOO. Samples presented to the assessors were randomly coded. Sensory attributes were quantified using an unstructured 10 cm intensity ordinal scale ranging from 0 (no perception) to 10 (highest intensity of perception). In addition, an evaluation sheet expanded with particular positive sensory attributes (“olive fruitiness“, “other ripe fruits“, “apple“, “green grass/leaves“, “sweet”) was used during the sensory analysis to better explain certain changes in the sensory profiles of the investigated VOOs. The overall sensory quality was graded using a rating scale from 1 (lowest quality) to 9 (highest quality).

### 2.6. Analysis of Volatile Compounds

The volatile composition of the VOOs was determined according to the method described by [31] using headspace solid-phase microextraction with gas chromatography/mass spectrometry (HS-SPME-GC/MS). GC/MS analyses were performed using a Varian 3900 gas chromatograph coupled to a Varian Saturn 2100T ion trap mass spectrometer (Varian Inc., Crawley, UK). Volatile compounds were identified by comparing their mass spectra with those of pure standards and with mass spectra from the NIST05 library. In addition, the identification of twenty volatile compounds was performed by comparing their retention times with those of pure standards. The standards were purchased from Aldrich (Steinheim, Germany), Fluka (Buchs, Germany), and Merck KGaA (Darmstadt, Germany). Linear retention indexes (LRI) were determined by calculation based on the retention times of a homologous series of linear alkanes (C7-C24) as a standard mixture in pure dichloromethane and compared with the LRIs available in the literature. Quantification was based on calibration curves of standards dissolved in fresh refined sunflower oil, while semi-quantitative analysis was performed for other volatile compounds. Their concentrations were expressed as equivalents of the compounds with similar chemical structures for which standards were available, assuming a response factor of one. 

### 2.7. Elaboration of the Data

Sensory and chemical analysis data were subjected to one-way analysis of variance (ANOVA) and mean values were compared using Tukey’s honest significant difference test at the *p* < 0.05 level. Pearson correlation coefficients (*r*) were calculated to assess the relationship between the degree of olive fruit infestation, the intensity of sensory defects in VOO samples and the concentrations of volatile compounds in VOO. ANOVA and correlation analysis were performed using Statistica v. 13.2 software (Stat-Soft Inc., Tulsa, OK, USA). The data were further processed with a hierarchical cluster analysis (HCA) and partial least squares discriminant analysis (PLS-DA) using MetaboAnalyst v. 5.0 software [32].

## 3. Results and Discussion

### 3.1. Oil Content and VOO Quality Parameters

The olive fly infestation did not cause significant changes in the water and oil content of the olive pastes (Table 1). This was not to be expected, as damaging infestations (presence of at least one exit hole) predominated over active infestations (presence of punctures). A partial explanation could be the relatively early ripening stage of the fruits (RI = 3.0), as Tamendjari et al. [17] showed that the oil content decreased significantly only in fruits infested by the olive fly at advanced ripening stages (RI higher than 3.5). 

Considering the level of FFA, a slight, albeit statistically significant, increase was determined only in L-100% VOO (Table 1). Olive fly attack may have enabled oil–water contact in olive fruits, which allowed exogenous and endogenous lipases to initiate the hydrolytic degradation of triglycerides, leading to an increase in FFA, as previously shown [33]. In terms of oxidation parameters, only PV was significantly increased in L-100% VOOs, probably due to ruptures of the olive fruit skin and a higher exposure of fatty acids in the attacked drupes to oxygen [15]. There is evidence that olive fruit fly infestation negatively affects the chemical quality of olives, but the degree of impact depends on the olive cultivar, the stage of ripeness of the infested fruits, and the type and severity of the infestation [15,18,33]. In this study, these impacts were found to be minimal, as the basic chemical parameters measured in all VOOs studied were within the legal limits for the extra virgin category [29]. This probably reflects the earlier stage of ripeness of the infested fruits, when the solid cellular structure of the mesocarp allowed only limited contact of air and water with the oil inside the drupes.

**Table 1:** Moisture and oil content based on dry weight in the olive paste and quality parameters (free fatty acids—FFA; peroxide value—PV; spectrophotometric indices—K_232_, K_270_, and ∆K) of virgin olive oils from fruits of the Leccino (L) cultivar with different levels of olive fly infestation (0%, 50%, and 100%).

Parameter	L-0%	L-50%	L-100%	EVOO *
Water in olive paste (%)	46.74 ± 1.03	46.63 ± 1.31	44.60 ± 0.24	
Oil in olive paste (%)	34.89 ± 1.24	36.67 ± 1.03	35.71 ± 2.89	
FFA (% oleic acid)	0.23 ± 0.01 ^b^	0.23 ± 0.00 ^b^	0.25 ± 0.00 ^a^	≤0.80
PV (mmol O_2_/kg)	7.55 ± 0.08 ^b^	7.61 ± 0.03 ^b^	10.76 ± 0.11 ^a^	≤20.0
K_232_	2.20 ± 0.09	2.18 ± 0.04	2.27 ± 0.08	≤2.50
K_270_	0.18 ± 0.00	0.17 ± 0.01	0.16 ± 0.00	≤0.22
ΔK	0.00 ± 0.00	0.00 ± 0.00	0.00 ± 0.00	≤0.01
Sensory score (1–9)	7.81 ± 0.48 ^a^	7.09 ± 0.74 ^b^	5.78 ± 0.39 ^c^	

The results for each infestation level are given as means ± standard deviation of three independent replicates. Means within a row marked with different letters, are significantly different (Tukey’s test, *p* ˂ 0.05). * Official limits for the extra virgin olive oil (EVOO) category [34].

### 3.2. Fatty Acid Composition

Olive fly infestation did not significantly affect the profile of fatty acids (Table 2), which is consistent with several previous studies [17,18,22,35]. On the other hand, some authors reported a negative correlation between the content of unsaturated fatty acids, especially oleic acid, and the degree of olive fly infestation in olive cultivars from Algeria [23]. Different effects of olive fly infestation on the composition of fatty acids in different studies were possibly a consequence of the different intensity and severity of the infestation, as Valenčič et al. [36] reported for olive oils from Istrska bjelica fruits. The same authors [36] found a lower content of oleic acid and a slightly higher content of myristic, linoleic, and linolenic acid as a result of heavy olive fly infestation (presence of emergence holes) compared to active infestation (presence of olive fly punctures). In this study, despite the predominant proportion of fruits with damaging infestation, only a low level of oxidation was achieved, as confirmed by the values of the quality parameters presented in Table 1. Moreover, the high ratio of oleic to linoleic acid (>11, Table 2) probably favoured the oxidative stability [37] of the VOOs studied.

### 3.3. Sensory Characteristics

**Figure 1:**
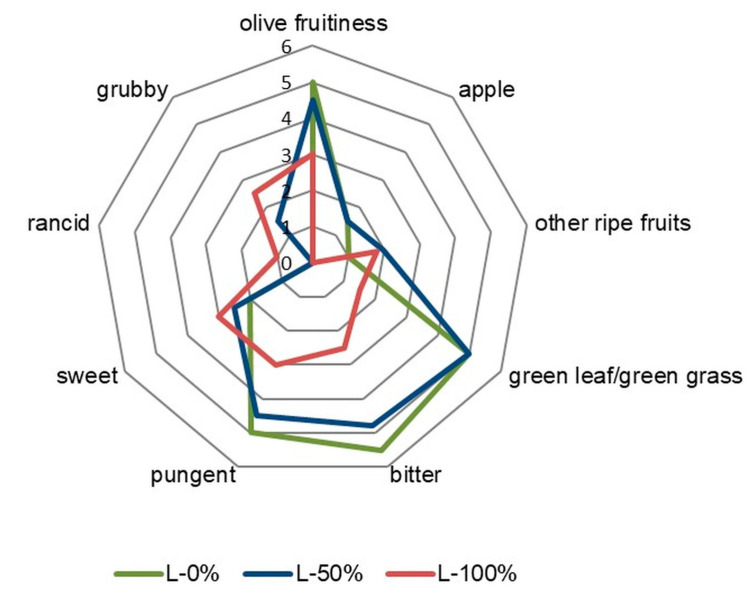
Sensory characteristics of virgin olive oils from fruits of the Leccino (L) cultivar with different levels of olive fly infestation (0%, 50%, and 100%). Results for each infestation level are mean values of 3 independent replicates.

Extra virgin olive oils (EVOO) are generally characterized by their fruitiness and the absence of any negative sensory characteristics, also known as sensory defects. Many factors can negatively affect the sensory quality of olive oil, including olive fly infestation [2]. The sensory characteristics of the Leccino VOOs obtained in this study are shown in Figure 1.

Among the positive olfactory characteristics of the VOOs, the intensity of “other ripe fruits” increased in oils from infested fruits (L-50% and L-100%), while the intensity of “olive fruitiness”, “apple” and “green leaf/green grass” decreased, especially in L-100%. The taste characteristics bitterness and pungency also showed a decreasing trend, especially in the case of L-100% VOOs. Bitterness and pungency are mainly related to the phenolic compounds of the olive oil [38]. Notario et al. [19] reported that the phenolic compound content of oils obtained from olive fly-infested fruits decreased as part of a biological response to stress following pathogen infestation. A decrease in the positive VOO sensory characteristics as a consequence of different olive fly infestation levels was also observed in previous studies on other cultivars [17,18,21,22].

Among the detected off-flavours, the most intense one in both Leccino VOOs from infested fruits was “grubby”, with a greater intensity in L-100% samples. The panel members described the “grubby” defect as reminiscent of cocoa butter flavour, fatty, and sweet. As no other major defects were found, the described “grubby” defect could be considered characteristic of oils obtained from olives that are significantly infested with olive fruit fly grubs. To our knowledge, only two studies have been published so far in which the “grubby” defect was found, but always in combination with some other defects [17,23]. In all available previous reports, including the two mentioned, the olive fly infestation was primarily associated with off-flavours resulting from fermentation processes (“fusty“ or “musty”) and oxidation (“rancid”), which makes the results of this study, with “grubby” as a predominant defect, quite unique. The low intensity of rancidity found in L-100% VOO was indicative of the onset of oxidative changes, which corresponds to the increased value of PV as an oxidation parameter in this sample (Table 1). All the mentioned negative changes in the sensory profile of the VOOs led to a decrease in the overall sensory scores, especially in L-100% samples (Table 1).

### 3.4. Volatile Composition

The volatile compounds found in the VOOs studied are listed in Table 3, sorted by descending value of the linear correlation coefficient between their concentrations and the degree of olive fly infestation. About twenty compounds showed a very strong positive linear correlation with the degree of infestation, as well as with the intensity of the “grubby” defect. Among them are ethyl 2-methylbutyrate, propanoic acid, dimethyl sulfoxide, 2-metylbutanal, 3-methylbutanal, and methyl acetate, which are generally associated with fermentative processes. The extent of fermentative changes inside olive fruit infested by the olive fly is probably related to the extent of damage and would be more pronounced if the olive fly had already created exit holes. It should be mentioned again that in this study a batch of olives infested by olive fly infestatio was represented by a mixture of fruits with a puncture or an exit hole. Prolonged storage time and unsuitable storage conditions of damaged fruits, which were avoided in this study, further contribute to the development of microbial populations and fermentative changes.

The concentration of ethyl 2-methylbutyrate, an ester formed by formal condensation of 2-methylbutyric acid with ethanol, increased in VOOs obtained from infested fruits. This compound was not only highly correlated with the degree of infestation, but also had nearly the highest correlation coefficient (*r*) with the intensity of the “grubby” defect.

Microbial fermentation of carbohydrates regularly produces organic acids, but in this study only the level of propanoic acid in L-100% VOO increased. Propanoic acid is often associated with pungent, sour, and mouldy flavours and has previously been found in elevated amounts in olive oils with the “fusty” defect [8,39]. In this experiment, “fusty” was not observed, most probably due to the very fast processing of the fruits after harvest. The concentration of this acid in the VOOs studied correlated very strongly with the intensity of the two defects “rancid” and “grubby”, as well as with the degree of infestation.

The concentration of dimethyl sulfoxide, a compound characterized by a garlic-like flavour [40], increased about twofold in L-50% and fivefold in L-100% compared to L-0% VOO. It showed a very strong correlation with the degree of infestation level and with the intensity of “rancid” and “grubby” defects (Table 3). Studies have found that dimethyl sulfide, a precursor of dimethyl sulfoxide, can be formed by yeasts from cysteine or glutathione during the fermentation of grape must [41] and contributes to the green olive aroma of wine [42]. Its increase in virgin olive oil could be due to the enzymatic activity of yeasts naturally present on the skin of olive fruits [43] and transferred to the olive endocarp during olive fly oviposition or through the exit holes of larvae. As reported by Guerrini et al. [44], yeasts associated with olives can affect the volatile composition and quality of the oil due to their metabolic activities and can be a cause of sensory defects of the oil [45].

An increase in 2-methylbutanal concentration in VOOs obtained from infested olives could also be related to yeast activity, since this branched aldehyde is a known metabolite of, e.g., *Saccharomyces cerevisiae* [46]. It has been characterized by a malty aroma [47], but has also been associated with fusel oils, cocoa, chocolate, almond, and cheese flavours, as well as “fusty” and “musty” defects [5,7]. 3-Methylbutanal, which is naturally produced by the microbial degradation of the amino acid leucine, is found in many foods, such as cheese, chocolate, fish, tea, and olive oil [48]. It has an odour described as malty, ethereal, aldehydic, chocolate, peach, or fatty, and was previously associated with a “fusty” defect [5]. Compared to L-0%, the concentration of both aldehydes was about twice and three times higher in L-50% and in L-100% VOO, respectively. It is possible that the increased concentrations of these compounds are related to the presence of fatty and cocoa butter odours perceived by the tasters during the sensory analysis, while they described the “grubby” defect in L-50% and L-100% oil samples.

Methyl acetate, which is often associated with the “winey-vinegary” oil defect [49], is more strongly correlated with the intensity of the defect “rancid” than with the intensity of the defect “grubby” or the degree of infestation (Table 3). It increased only very slightly and only in L-100% VOO, probably as a consequence of the enzymatic activities of moulds and yeasts after olive fly infestation. For instance, Guerrini et al. [44] found that the concentration of methyl acetate in VOOs correlated positively with the concentration of yeasts inoculated on olive fruits from which the VOOs were obtained.

Damage to the cell structure of olive fruit by the olive fly can lead to fermentation and the conversion of sugars to ethanol, acetic acid, and ethyl acetate, which are strongly associated with the “fusty” defect in olive oil [9]. Notario et al. [19] and Angerosa et al. [16] found an increase in ethanol and ethyl acetate levels in oils from infested olive fruits. Tamendjari et al. [17] also found an increase in the concentration of acetic acid and various alcohols, such as methanol, ethanol, and 3-methyl-1-butanol, all of which were found in oils with the defect “winey” [9]. In this study, no statistically significant change in ethyl acetate concentration was found in relation to olive fly infestation, while changes in acetic acid concentration were not linearly related to the degree of infestation, as the lowest value was recorded in L-50% VOO (Table 3).

Several volatile compounds associated with olive oil oxidation were determined in VOOs obtained from fruits infested by the olive fly. The levels of 4-methyl-5H-furane-2-one, 6-methyl-5-hepten-2-one, octanal, hexanal, heptanal, *E*-2-nonen-1-ol, 1-octanol, *E*-2-heptenal, benzeneacetaldehyde, and *E,E*-2,4-heptadienal showed a very strong correlation with the degree of infestation (Table 3). These compounds were probably formed in higher amounts because the olive fatty acids were more exposed to atmospheric oxygen due to the fruit lesions caused by the olive fly.

Compared to L-0% VOO, the level of 4-methyl-5H-furane-2-one increased about fivefold in L-50% and eightfold in L-100% VOO, while the content of 6-methyl-5-hepten-2-one increased about twofold in L-50% and about threefold in L-100% VOO. Bendini et al. [50] reported an association between increased 6-methyl-5-hepten-2-one levels and the degree of olive fly infestation, whereas Guerrini et al. [44] found an increase in olive oils from fruits inoculated with yeasts. This unsaturated methylated ketone, whose odour is described as oily, herbaceous, green fruity, and pungent, has previously been associated with the defect “rancid” by several authors [9,39,51], while it has also been associated with the defect “frostbitten olives” [51].

Medium-chain aldehydes, such as hexanal and octanal, have shown a similar trend but with a less-pronounced increase in concentration than for the aforementioned ketones. An increase in hexanal concentration in oil samples from infested olive fruits was also found by Angerosa et al. [16] and Brkić Bubola et al. [18]. Both above-mentioned aldehydes were characterized by pleasant odour descriptions at low concentrations (green, cut grass, apple, leaf, citrus), while high concentrations can lead to the defect “rancid”, evoking sensations described as fatty, soap [51], oily, greasy, and woody [9,11,39]. In this experiment, the concentrations of all the compounds mentioned correlated more strongly with the intensity of “grubby” than with that of “rancid”. The opposite was observed for unsaturated medium-chain aldehydes, such as *E*-2-heptenal and (*E*,*E*)-2,4-heptadienal. The odour of the former was previously described as oxidized, tallow, and pungent; that of the latter as fatty. Both were found in increased amounts in “rancid” oils by Neugebauer et al. [11], Cevik et al. [52], and Morales et al. [9]. In addition, Guerrini et al. [44] reported an increase in *E*-2-heptenal levels in olive oils obtained from fruits inoculated with yeasts.

Another compound with a very strong correlation with the degree of infestation and “rancid” and “grubby” defects was benzeneacetaldehyde (Table 3). According to the literature, it has a pleasant floral odour, sometimes reminiscent of bitter almonds, but also of rancid aromas [53]. Other authors described its odour as honey-like and beeswax-like [11]. It originates from the Strecker degradation of certain amino acids, such as leucine or phenylalanine, which is triggered by a wide range of lipid oxidation products [54]. Since peroxides are lipid oxidation products, the increase in benzeneacetaldehyde concentration by almost threefold in L-50% and sixfold in L-100% compared to L-0% VOO could be partially caused by the increase in fatty acid peroxides due to olive fly infestation (Table 1).

The concentration of ß-ocimene, an acyclic monoterpene characterized by green and floral notes [5], increased linearly. Concentrations in L-50% and L-100% were about four and seven times higher, respectively, compared to L-0% VOO, with a very strong positive correlation with the intensity of the defect “grubby” (Table 3). A four- to fivefold increase in ß-ocimene in oils from infested fruits was also found by Notario et al. [19], while Giunti et al. [55] also reported its increase in fruits infested by olive flies. According to Farré-Armengo et al. [56], plants infested by herbivores increase the emission of ß-ocimene from damaged and undamaged tissues as a systemic defence response. The concentration of the sesquiterpene α-farnesene also increased linearly, sixfold in L-50% and tenfold in L-100% compared to L-0% VOO. Notario et al. [19] found a similar increase in α-farnesene concentration as for ß-ocimene. α-Farnesene is associated with woody, herbaceous, citrus, and floral aromas [7]. Previously, α-farnesene was considered to be a pheromone of the Mediterranean fruit fly *Ceratitis capitata* (Ceratitidini), released by mature males [57], and possibly it has the same function in the metabolism of the olive fly. Therefore, a transfer of this substance from olive fruits infested by the olive fly to the oil was considered plausible.

As for the effects of olive fly infestation on the volatile products of the LOX pathway, several authors have reported particular changes, albeit with different and contradictory results. Tamendjari et al. [17], Bendini et al. [50], and Brkić Bubola et al. [18] found a decrease in the levels of *E*-2-hexenal, the most abundant product of the LOX pathway, in oil samples from fully infested fruits. On the other hand, Angerosa et al. [16], Alagna et al. [20], and Notario et al. [19] reported an increase as a result of plant–insect interaction, which is consistent with the results of Landa et al. [58], who studied oils from olive trees infected by the fungal plant pathogen *Verticillium dahlia*. In this experiment, the levels of *E*-2-hexenal and all other identified C6 aldehydes (Z-2-hexenal, *E*-2-hexenal, *Z*-3-hexenal, *E*-3-hexenal, *E,Z*-2,4-hexadienal, *E,E*-2,4-hexadienal) were not positively correlated with the degree of infestation, except for hexanal. All the above-mentioned compounds showed a very similar trend in concentration variations in response to olive fly infestation, with a minimum in L-50% VOO. Given their known contribution to olive oil fruitiness [7,59], these results are unexpected and inconsistent with the sensory characteristics of VOOs obtained from partially (L-50%) and fully infected fruits (L-100%) (Figure 1). However, Angerosa et al. [16] found that despite an increase in *E*-2-hexenal content, the value of the *E*-2-hexenal/hexanal ratio decreased progressively with an increase in olive fly infestation degree. This was confirmed in this study, as the *E*-2-hexenal/hexanal ratio decreased from 157.9 (L-0%) to 58.3 (L-50%) and 99.8 (L-100%), indicating a lower quality of the oils extracted from infested fruits.

Interestingly, there is a consistent trend within the group of C5 alcohols formed via the LOX pathway (1-pentanol, *E*-2-penten-1-ol, *Z*-2-penten-1-ol, and 1-penten-3-ol) that show a maximum in L-50% VOOs, which is the opposite direction to that of C6 aldehydes. Moreover, Notario et al. [19] reported that olive oils obtained from fruits of the Picual, Manzanilla, and Hojiblanca cultivars infested by olive fruit fly had a decreased content of 1-penten-3-one, which was confirmed by the results of the present study (Table 3). 1-Penten-3-one contributes to pungent sensory notes in olive oil and, due to a low threshold, could have a significant impact on the olive oil aroma [59]. In fact, one of the reasons for the lower pungency in VOOs obtained from infested fruits (Figure 1) could be the lower 1-penten-3-one concentration in these samples (Table 3).

As regards the identified C6 alcohols from the LOX pathway, no consistent trend was observed. Some of them, such as 1-hexanol and *E*-2-hexen-1-ol, showed a moderate or strong positive correlation, while others, such as *E*-3-hexen-1-ol and *Z*-3-hexen-1-ol, showed a negative correlation with the degree of infestation.

As mentioned above, in previous studies, a “grubby” defect was rarely found in olive oils extracted from fruits infested by the olive fly. So far, no connection has been found between this defect and volatile compounds that could be responsible for its occurrence. According to the results discussed above, it seems that infestation by the olive fly under relatively favourable conditions (i.e., when the fruit is at a relatively early stage of ripeness and the fruit is stored for a very short time) causes oxidative changes accompanied by slight fermentative degradation of the olive oil. To assess the suitability of volatile compounds as markers of the presence and intensity of the defect “grubby”, the Pearson correlation coefficient (*r*) was used to measure the strength of the linear relationship between the concentration of a given VOO volatile compound and the intensity of the defect. Among compounds related to oxidative degradation, the highest positive correlation with the defect “grubby” was found for 1-octanol (*r* = 0.9998), octanal (*r* = 0.9965), 4-methyl-5H-furan-2-one (*r* = 0.9944), hexanal (*r* = 0.9922), and 6-methylen-5-hepten-2-one (*r* = 0.9904), followed by *E*-2-nonen-1-ol, heptanal, and *E*-2-heptenal. Among the compounds associated with fermentative degradation, those most positively correlated with the intensity of the “grubby” defect were ethyl 2-methylbutyrate (*r* = 0.9992) and propanoic acid (*r* =0.9491), followed by benzeneacetaldehyde, dimethyl sulfoxide, 2-methylbutanal, and 3-methylbutanal. In a group of volatiles of other origin, α-farnesene (*r* = 0.9973) and *ß*-ocimene (*r* = 0.9924) stood out, followed by phenylethyl alcohol. These compounds could be considered as having the greatest potential to serve as characteristic markers for the occurrence of the “grubby” defect in olive oil. On the other hand, the most negative correlation with the intensity of the “grubby” defect in the studied VOOs was found for branched-chain alkene II (*r* = −0.9973), 3-ethyl-1,5-octadiene I (*r* = −0.9899), pentanoic acid (*r* = −0.9895), 3-ethyl-1,5-octadiene II (*r* = −0.9833), and phenol (*r* = −0.9806).

### 3.5. Hierarchical Clustering Analysis (HCA)

**Figure 2:**
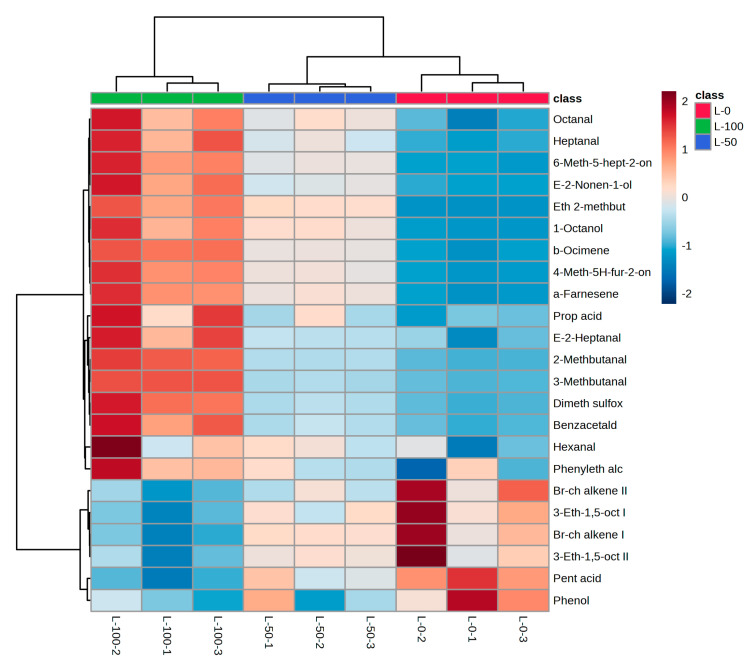
Clustering of virgin olive oils from fruits of the Leccino (L) cultivar with different levels of olive fly infestation (L-0: 0% infestation; L-50: 50% infestation; L-100: 100% infestation) according to hierarchical cluster analysis (HCA) based on the composition of selected volatile compounds as variables. The rows in the heatmap diagram represent volatile compounds and the columns represent samples. The sample codes were formed according to the following principle: Leccino (L) olive oil from olives with 0% (0) olive fruit fly infestation in the first replicate (1) was coded as L-0-1 and so on. The colours of the heatmap cells indicate low (dark blue), medium (white), and high (dark red) abundance of a particular volatile compound.

In order to better identify certain volatile compounds characteristic of oils extracted from olives with different levels of olive fruit fly infestation, a hierarchical clustering analysis (HCA) was conducted with the studied Leccino VOOs grouped into three groups according to the degree of olive fruit fly infestation. The variables used were the volatile compounds for which the highest positive (*r* > 0.9) or negative (*r* < −0.9) correlations with the intensity of the “grubby” defect were found (Table 3). The groups were successfully clustered, as shown in a heatmap diagram in Figure 2.

The three groups studied were mainly related to the volatile compounds characteristic of each group previously determined with ANOVA (Table 3). VOOs from olives with 100% infestation (L-100%) were separated from the others by the highest Euclidean distances and were characterized by higher levels of several volatile compounds, including certain aldehydes, ketones, and esters, as well as certain sulphur, furanoid, and benzenoid compounds of fermentative, oxidative, and other origin, with the exception of the LOX pathway and terpenoids, which are probably released in greater quantities as part of the defence of olives against external pathogens. VOOs from olives with 50% infestation (L-50%) contained intermediate concentrations of most of the compounds included, while VOOs from healthy fruits (L-0%) were clustered together and separated from the others, as they contained lower concentrations of the previously mentioned compounds, as well as higher concentrations of several unsaturated branched-chain alkenes, which are often found in higher levels in higher-quality olive oils [60,61]. Major LOX-derived C6 and C5 compounds were not included in the model due to their irregular behaviour and weaker correlation with the intensity of the defect “grubby”.

**Figure 3:**
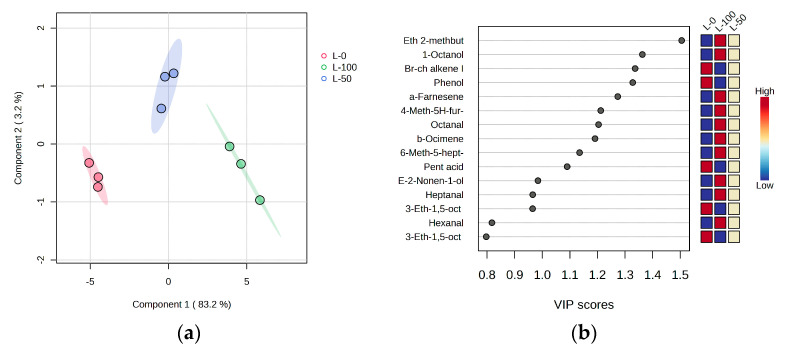
(**a**) Separation of virgin olive oils from fruits of the Leccino (L) cultivar with different levels of olive fly infestation (L-0: 0% infestation; L-50: 50% infestation; L-100: 100% infestation) in two-dimensional space according to partial least squares discriminant analysis (PLS−DA); (**b**) variable importance in projection (VIP) scores of selected volatile compounds as variables most useful for the separation.

The data were further subjected to partial least squares discriminant analysis (PLS-DA; Figure 3). The separation by degree of infestation was very good and the three groups were successfully separated. The variables with the highest variable importance in projection (VIP) scores (VIP > 1.2), and therefore most useful for separation, were ethyl 2-methylbutyrate, 1-octanol, α-farnesene, 4-methyl-5H-furan-2-one, octanal, and β-ocimene, which were most abundant in L-100% VOOs, as well as branched-chain alkene I and phenol, which are characteristic for L-0% VOOs. Intermediate levels of all 15 volatile compounds most useful for separation were found in L-50% VOOs.

**Figure 4:**
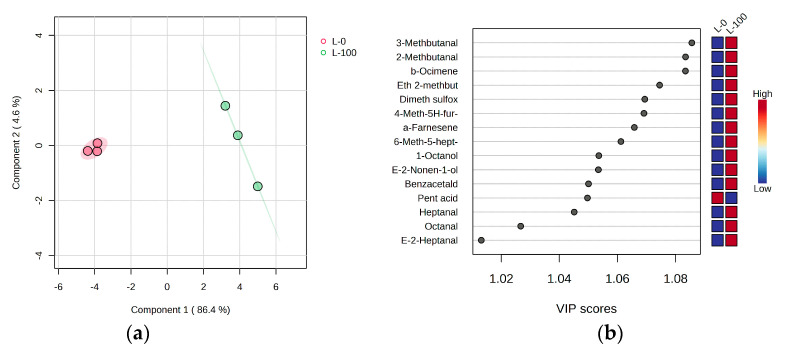
(**a**) Separation of virgin olive oils from fruits of the Leccino (L) cultivar with different levels of olive fly infestation (L-0: 0% infestation; L-100: 100% infestation) in two-dimensional space according to partial least squares discriminant analysis (PLS−DA); (**b**) variable importance in projection (VIP) scores of selected volatile compounds as variables most useful for the separation.

With the aim of additionally filtering out potential volatile markers for the occurrence and intensity of the “grubby” defect, PLS-DA was repeated, but this time only two extreme groups were included, namely those of VOOs produced from healthy (L-0%) and fully infested fruits (L-100%), which were characterized by the absence or highest intensity of the “grubby” defect. Again, the separation was successful (Figure 4a), while the volatiles with the highest discriminatory power, i.e., the highest VIP scores, were 3-methylbutanal, 2-methylbutanal, β-ocimene, ethyl 2-methylbutyrate, dimethyl sulfoxide, 4-methyl-5H-furan-2-one, α-farnesene, and 6-methyl-5-hepten-2-one, followed by others, mostly aldehydes, almost all characteristic of L-100% VOOs with an intense “grubby” defect.

## 4. Conclusions

In this study, processing olive fruits infested by the olive fruit fly at an early stage of ripeness and without prior storage allowed us to obtain virgin olive oils with “grubby” as the main perceived sensory defect. Without major interferences from other defects often associated with olive fruit fly infestation, such as “fusty”, “musty”, or “rancid”, it was possible to define more precisely its sensory descriptors (such as cacao butter-like, fatty, and sweet) and to relate their intensity to the degree of olive fly infestation. The occurrence and intensity of “grubby” was clearly causally related to the concentrations of certain volatile compounds, although particularly those often listed as products of fermentative or oxidative degradation. In addition, olive fly infestation increased the levels of certain terpenes that are regularly emitted by plant tissues as a systemic defence response to attack by pathogens or parasites. Based on Pearson correlation and partial least squares discriminant analyses, several compounds were singled out as chemical markers. The highest potential impact was attributed to 3-methylbutanal, 2-methylbutanal, β-ocimene, ethyl 2-methylbutyrate, dimethyl sulfoxide, 4-methyl-5H-furan-2-one, α-farnesene, 6-methyl-5-hepten-2-one, 1-octanol, *E*-2-nonen-1-ol, benzeneacetaldehyde, heptanal, and octanal, implying that the perception of “grubby” derived from their joint contribution to the overall aroma of the olive oil. The results obtained could contribute to the development of strategies for the instrumental support of sensory analysis in the classification of olive oil quality and the confirmation of the presence of defects in olive oil. In addition to contributing to the understanding of the chemical origin of the “grubby“ defect in olive oils, further research on other cultivars is recommended to obtain a larger database of volatiles associated with the “grubby” defect.

## Figures and Tables

**Table 2:** Fatty acid composition (%) of virgin olive oils from fruits of the Leccino (L) cultivar with different levels of olive fly infestation (0%, 50%, and 100%).

Fatty Acid	L-0%	L-50%	L-100%	EVOO *
Myristic (C 14:0)	0.01 ± 0.00	0.01± 0.00	0.01 ± 0.00	≤0.03
Palmitic (C 16:0)	12.81 ± 0.29	12.77 ± 0.26	13.92 ± 1.04	7.50–20.00
Palmitoleic (C 16:1)	1.31 ± 0.05	1.21 ± 0.07	1.21 ± 0.07	0.30–3.50
Heptadecanoic (C 17:0)	0.03 ± 0.00	0.03 ± 0.00	0.03 ± 0.00	≤0.40
Heptadecenoic (C 17:1)	0.06 ± 0.00	0.06 ± 0.00	0.07 ± 0.01	≤0.60
Stearic (C 18:0)	2.05 ± 0.01	2.04 ± 0.02	1.99 ± 0.00	0.50–5.00
Oleic (C 18:1)	75.66 ± 0.28	75.85 ± 0.32	74.61 ± 1.00	55.0–83.0
Linoleic (C 18:2)	6.54 ± 0.01	6.54 ± 0.03	6.50 ± 0.15	2.50–21.00
Linolenic (C18:3)	0.39 ± 0.01	0.38 ± 0.01	0.37 ± 0.01	≤1.00
Arachidic (C 20:0)	0.63 ± 0.00	0.66 ± 0.01	0.71 ± 0.04	≤0.60
Eicosenoic (C 20:1)	0.32 ± 0.01	0.31 ± 0.01	0.31 ± 0.04	≤0.50
Behenic (C 22:0)	0.13 ± 0.01	0.12 ± 0.00	0.11 ± 0.01	≤0.20
Erucic (C 22:1)	0.00 ± 0.00	0.00 ± 0.00	0.02 ± 0.01	
Lignoceric (C 24:0)	0.07 ± 0.00	0.06 ± 0.00	0.07 ± 0.00	≤0.20
Oleic/linoleic ratio (C18:1/C18:2)	11.57 ± 0.02	11.60 ± 0.00	11.49 ± 0.11	

The results for each infestation level are given as means ± standard deviations of three independent replicates. Means within a row marked with different letters, are significantly different (Tukey’s test, *p* ˂ 0.05). * Official limits for the extra virgin olive oil (EVOO) category [34].

**Table 3:** Volatile compounds (mg/kg) in virgin olive oils from fruits of the Leccino (L) cultivar with different levels of olive fly infestation (0%, 50%, and 100%) and correlation coefficients between the concentration of volatile compounds and the intensity of sensory defects or the level of infestation of the olive fruits.

Volatile Compounds	L-0%	L-50%	L-100%	Correlation (*r*) with Intensity of:		
				“Rancid”	“Grubby”	Infestation
4-Methyl-5H-furan-2-one *	0.022 ± 0.005 ^c^	0.106 ± 0.003 ^b^	0.188 ± 0.025 ^a^	**0.8613**	**0.9944**	**1.0000**
*ß*-Ocimene	0.027 ± 0.007 ^c^	0.112 ± 0.001 ^b^	0.200 ± 0.008 ^a^	**0.8701**	**0.9924**	**0.9999**
6-Methyl-5-hepten-2-one	0.146 ± 0.005 ^c^	0.281 ± 0.006 ^b^	0.427 ± 0.053 ^a^	**0.8775**	**0.9904**	**0.9997**
Octanal	0.052 ± 0.006 ^c^	0.075 ± 0.003 ^b^	0.096 ± 0.012 ^a^	**0.8501**	**0.9965**	**0.9996**
*α*-Farnesene *	0.011 ± 0.003 ^c^	0.066 ± 0.002 ^b^	0.114 ± 0.017 ^a^	**0.8444**	**0.9973**	**0.9992**
Hexanal	0.028 ± 0.004	0.032 ± 0.002	0.037 ± 0.007	**0.8711**	**0.9922**	**0.9979**
Heptanal *	0.117 ± 0.011 ^c^	0.248 ± 0.019 ^b^	0.431 ± 0.076 ^a^	**0.9099**	**0.9779**	**0.9954**
Ethyl 2-methylbutyrate	0.000 ± 0.000 ^c^	0.003 ± 0.000 ^b^	0.005 ± 0.001 ^a^	0.7790	**0.9992**	**0.9934**
*E*-2-Nonen-1-ol *	0.006 ± 0.000 ^c^	0.010 ± 0.000 ^b^	0.016 ± 0.002 ^a^	**0.9080**	**0.9788**	**0.9934**
1-Octanol *	0.010 ± 0.000 ^c^	0.015 ± 0.000 ^a^	0.018 ± 0.002 ^b^	**0.8146**	**0.9998**	**0.9897**
*E*-2-Heptenal *	0.377 ± 0.061 ^b^	0.462 ± 0.005 ^a^	0.692 ± 0.086 ^a^	**0.9652**	**0.9309**	**0.9664**
Propanoic acid *	0.016 ± 0.001 ^b^	0.017 ± 0.001 ^b^	0.020 ± 0.002 ^a^	**0.9498**	**0.9491**	**0.9608**
Benzeneacetaldehyde	0.046 ± 0.009 ^b^	0.101 ± 0.008 ^b^	0.269 ± 0.050 ^a^	**0.9715**	**0.9214**	**0.9598**
Dimethyl sulfoxide *	0.087 ± 0.009 ^c^	0.166 ± 0.008 ^b^	0.424 ± 0.051 ^a^	**0.9744**	**0.9163**	**0.9560**
2-Methylbutanal *	0.005 ± 0.001 ^c^	0.010 ± 0.000 ^b^	0.028 ± 0.001 ^a^	**0.9749**	**0.9156**	**0.9507**
3-Methylbutanal	0.005 ± 0.001 ^c^	0.011 ± 0.001 ^b^	0.033 ± 0.000 ^a^	**0.9822**	**0.9007**	**0.9496**
Phenylethyl alcohol	0.019 ± 0.003 ^b^	0.020 ± 0.001 ^a^	0.024 ± 0.002 ^a^	**0.9449**	**0.9538**	**0.9449**
*E*,*E*-2,4-Heptadienal *	0.465 ± 0.094 ^b^	0.490 ± 0.022 ^b^	0.755 ± 0.065 ^a^	**0.9970**	**0.8467**	**0.9023**
Methyl acetate *	0.097 ± 0.006 ^b^	0.098 ± 0.002 ^b^	0.111 ± 0.001 ^a^	**0.9984**	**0.8358**	**0.8963**
1,3,5,5-Tetramethyl-1,3-c *	0.135 ± 0.177	0.140 ± 0.007	0.248 ± 0.079	**0.9992**	**0.8256**	**0.8875**
Butyric acid	0.006 ± 0.002	0.007 ± 0.000	0.007 ± 0.000	**0.9895**	**0.8808**	**0.8660**
Hexanoic acid *	0.020 ± 0.005	0.026 ± 0.009	0.024 ± 0.003	0.1666	0.7215	0.6547
3,7-Decadiene III *	0.049 ± 0.010	0.063 ± 0.003	0.055 ± 0.006	−0.0947	0.5173	0.6423
1-Hexanol	0.108 ± 0.048	0.146 ± 0.002	0.130 ± 0.011	0.0935	0.6685	0.5765
Ethyl acetate *	0.046 ± 0.046	0.029 ± 0.001	0.068 ± 0.009	**0.9071**	0.4775	0.5626
3,7-Decadiene II *	0.094 ± 0.020	0.119 ± 0.012	0.104 ± 0.002	−0.1058	0.5078	0.3974
1-Pentanol	0.018 ± 0.003 ^c^	0.070 ± 0.001 ^a^	0.038 ± 0.003 ^b^	−0.1319	0.4850	0.3812
*E*-2-Hexen-1-ol	0.426 ± 0.451 ^b^	3.069 ± 0.118 ^a^	0.926 ± 0.031 ^b^	−0.3379	0.2897	0.1780
*E*-2-Penten-1-ol	0.047 ± 0.003 ^c^	0.101 ± 0.003 ^a^	0.057 ± 0.004 ^b^	−0.3380	0.2896	0.1741
*Z*-2-Penten-1-ol	0.159 ± 0.010 ^b^	0.203 ± 0.008 ^a^	0.167 ± 0.015 ^b^	−0.3475	0.2798	0.1706
Benzyl alcohol	0.001 ± 0.000	0.001 ± 0.000	0.001 ± 0.000	**−0.9943**	**−0.8618**	**0.0000**
*E*-2-Pentenal	0.018 ± 0.002 ^a^	0.007 ± 0.001 ^b^	0.017 ± 0.001 ^a^	0.4047	−0.2201	−0.0825
*Z*-2-Hexenal *	0.058 ± 0.010 ^a^	0.026 ± 0.001 ^b^	0.054 ± 0.010 ^a^	0.3832	−0.2429	−0.1149
Acetic acid *	0.859 ± 0.027 ^a^	0.337 ± 0.009 ^b^	0.786 ± 0.051 ^a^	0.3829	−0.2431	−0.1292
*E*-2-Hexenal	4.421 ± 0.610 ^a^	1.866 ± 0.052 ^b^	3.692 ± 0.338 ^a^	0.2406	−0.3853	−0.2769
*E*-3-Hexenal *	0.037 ± 0.008 ^a^	0.011 ± 0.001 ^b^	0.028 ± 0.007 ^a^	0.1896	−0.4330	−0.3409
*E*,*Z*-2,4-Hexadienal *	0.235 ± 0.023 ^a^	0.103 ± 0.001 ^c^	0.183 ± 0.022 ^b^	0.1196	−0.4957	−0.3910
*Z*-3-Hexenal *	0.055 ± 0.010 ^a^	0.021 ± 0.001 ^b^	0.041 ± 0.003 ^a^	0.1006	−0.5122	−0.4096
*E*-3-Hexen-1-ol	0.013 ± 0.012	0.018 ± 0.001	0.007 ± 0.000	**−0.9146**	−0.4933	−0.5447
*E*,*E*-2,4-Hexadienal *	0.070 ± 0.008 ^a^	0.023 ± 0.002 ^c^	0.042 ± 0.004 ^b^	−0.1159	−0.6851	−0.5921
1-Penten-3-ol	0.367 ± 0.034 ^ab^	0.394 ± 0.008 ^a^	0.327 ± 0.023 ^b^	**−0.9131**	−0.4901	−0.5933
1-Penten-3-one	0.470 ± 0.032 ^a^	0.035 ± 0.001 ^c^	0.196 ± 0.012 ^b^	−0.1479	−0.7083	−0.6229
*E*-2-Hexenoic acid *	0.019 ± 0.000 ^a^	0.009 ± 0.003 ^b^	0.010 ± 0.001 ^b^	−0.4527	**−0.8949**	**−0.8171**
*Z*-3-Hexen-1-ol	0.116 ± 0.080	0.107 ± 0.005	0.066 ± 0.004	**−0.9852**	**−0.8932**	**−0.9380**
Phenol *	0.387 ± 0.042	0.327 ± 0.044	0.310 ± 0.020	−0.6707	**−0.9806**	**−0.9517**
Branched-chain alkene I *	0.052 ± 0.008 ^a^	0.047 ± 0.000 ^ab^	0.037 ± 0.003 ^b^	**−0.9387**	**−0.9592**	**−0.9820**
Branched-chain alkene II *	0.045 ± 0.007 ^a^	0.036 ± 0.002 ^ab^	0.031 ± 0.003 ^b^	−0.7572	**−0.9973**	**−0.9867**
Pentanoic acid *	0.020 ± 0.001 ^a^	0.018 ± 0.001 ^b^	0.015 ± 0.001 ^c^	**−0.8806**	**−0.9895**	**−0.9934**
3-Ethyl-1,5-octadiene II *	0.188 ± 0.035	0.166 ± 0.002	0.138 ± 0.015	**−0.8981**	**−0.9833**	**−0.9976**
3-Ethyl-1,5-octadiene I *	0.239 ± 0.039 ^a^	0.203 ± 0.011 ^ab^	0.163 ± 0.014 ^b^	**−0.8793**	**−0.9899**	**−0.9995**

The results for each infestation level are given as means ± standard deviations of three independent replicates. Means within a row marked with different letters, are significantly different (Tukey´s test, *p* ˂ 0.05). * The volatile compounds for which pure standards were not available were quantified semi-quantitatively, and their concentrations were expressed as equivalents of the compounds with similar chemical structure for which standards were available, assuming a response factor = 1. *r*—Pearson correlation coefficient: 0.00–0.19 is considered very weak, 0.20–0.39 weak, 0.40–0.59 moderate, 0.60–0.79 strong, and 0.80–1.0 very strong correlation (in bold).

## Data Availability

Data are contained within the article.
